# Occupational Justice PLUS: From theoretical salience to practical application—An analysis of community narratives from a bushfire event

**DOI:** 10.1111/1440-1630.70131

**Published:** 2026-10-07

**Authors:** Eliza Huppatz, Carol McKinstry, Gail Whiteford, Emma George

**Affiliations:** ^1^ School of Allied Health and Human Performance Adelaide University Adelaide South Australia Australia; ^2^ Rural Department of Allied Health La Trobe University Bendigo Victoria Australia; ^3^ School of Allied Health, Sport and Exercise Sciences Charles Sturt University Port Macquarie New South Wales Australia

**Keywords:** disaster recovery, narrative analysis, occupational therapy, phenomenological research, social justice, wildfires

## Abstract

**Introduction:**

Emerging scholarship highlights the dominance of Western epistemologies in disaster research, limiting diversity and participation in knowledge production. The occupational therapy profession faces similar critiques of epistemic and hegemonic blind spots, prompting calls for critical reflection on professional assumptions. Drawing on narratives and guided by Whiteford's Occupational Justice PLUS model, we explore how learnings from community members' responses to a bushfire event could broaden our understanding of occupational, epistemic, and hermeneutic justice in the context of disasters and, more broadly, other injustices.

**Methods:**

This qualitative study used a phenomenological hermeneutical approach to explore narratives of disaster recovery. Data included a written narrative from ‘Doing Our Best’ and the film ‘We Are Conjola’. Analysis followed Lindseth and Norberg's three‐step process: naïve reading, structural analysis of themes, and comprehensive interpretation informed by literature and theories.

**Findings:**

Findings revealed interconnected injustices. Occupational injustice arose from restricted access to resources during bushfire response and recovery, with community‐led initiatives critical to restoring occupational justice. Epistemic injustice was evident in government disregard for climate science and Indigenous knowledge, compounded by media erasure, prompting the community to create its own narrative through film. Hermeneutic injustice occurred when aspects of social identity were obscured from collective understanding; residents responded through artistic expression to reclaim identity, share lived experience, and communicate systemic failures.

**Discussion:**

Community‐driven action addressed occupational, epistemic, and hermeneutic injustices following the Conjola bushfire. These findings highlight the relevance of the Occupational Justice PLUS model for disaster contexts, emphasising the need for occupational therapists to adopt critical, justice‐oriented approaches that privilege community voice and foster community‐led action in disaster recovery and knowledge creation.

**Conclusion:**

Advocacy for community voice and future research on applying the Occupational Justice PLUS model are essential to support inclusive, community‐driven recovery and strengthen knowledge creation in disaster contexts.

## PLAIN LANGUAGE SUMMARY

When disasters strike, communities often face challenges that go beyond physical damage. This study examined how people in Lake Conjola, New South Wales, responded after a major bushfire in 2019. We wanted to understand what their experiences could teach us about recovery after a disaster. We looked at community stories to learn what mattered most to residents. People described feeling abandoned during the fire and recovery, showing how hard it was to do everyday tasks without support. They were also frustrated that governments ignored warnings about climate change and traditional Indigenous land practices. Many felt their voices were silenced by the media. To take back control, the community created art, music, and film to share their story and push for change. These actions helped people heal and made sure their learnings were shared. For occupational therapists, we need to work with communities in ways that respect their knowledge and creativity. It also means sharing people's stories to make sure local voices are heard in disaster planning.

**Key Points for Occupational Therapy**

- Narrative and creative methods provide meaningful avenues for privileging community participation in knowledge creation and disaster recovery processes.
- Amplifying community stories strengthens advocacy efforts and supports policy development that enhances disaster preparedness and response.
- The Occupational Justice PLUS model offers a practical framework to understand justice‐oriented disaster recovery and critical reflection with implications for occupational therapy practice.

## 1 INTRODUCTION

The need to consider the impact of disasters from an occupational justice perspective has been well established in the literature (Carra, 2014; Carra et al., 2019; Huppatz et al., 2025; Rushford et al., 2015; Rushford & Thomas, 2016; Townsend & Marval, 2013). However, this study argues that a very narrow focus on occupational justice may potentially risk perpetuating the exclusion and marginalisation of subsections of the community. Simultaneously, scholars have increasingly called for the application of a critical approach to research in the disaster field, suggesting that hegemony of Western studies on disaster has privileged colonial and imperialist agendas, thereby imposing Western ontologies and epistemologies as common sense (Gaillard, 2022). This approach has resulted in the omission of crucial information due to a lack of participation, inclusion, and diversity in knowledge production (Khan et al., 2022).

Critiques of the occupational therapy profession have centred on epistemic and hegemonic ‘blindspots’, which have perpetuated the exclusion and marginalisation of community members whose worldviews may differ from the dominant discourse deeply rooted within the professions' knowledge base (Beagan et al., 2022; Córdoba, 2020; Farias & Rudman, 2019; Gerlach et al., 2018; Hammell, 2017; Hammell, 2021; Hammell & Iwama, 2012; Kinsella & Whiteford, 2009; Pooley & Beagan, 2021; Rudman, 2018; Whiteford, 2019). To address this, there has been a call to adopt a critical stance to reflect on and interrogate professional values, assumptions, ways of doing, and ways of knowing within the occupational therapy profession (Kinsella & Whiteford, 2009; Whiteford, 2019). Debate around social practices in occupational therapy, power, and justice has been prevalent in occupational therapy in the Global South, where in Brazil, occupational therapists have a long‐established response to experiences of colonisation and unequal distributions of power (Guajardo et al., 2015). Such a critical standpoint has begun to gain global momentum among scholars and practitioners (Gerlach, 2015; Kiepek et al., 2019). Gerlach (2015) called for a sharper critical edge in occupational therapy, and this seminal work has been cited over 100 times by scholars around the world and across disciplines (Emery‐Whittington, 2021; Jónasdóttir & Horghagen, 2025; Khalid & Bouakaz, 2025).

One attempt to operationalise such critical reflection within occupational therapy was the development of the Occupational Justice PLUS model by Whiteford (2019). The model was proposed as a framework for cultivating critical consciousness of gaps within the profession's knowledge systems by integrating three interconnected dimensions: ways of—and opportunities for—doing (occupational justice), ways of knowing (epistemic justice), and ways of being understood and represented (hermeneutic justice). Although first introduced in 2019, the model remains in its early stages of theoretical development, and there has been limited published exploration of its application. Accordingly, this paper does not seek to offer a definitive validation of the model but rather to contribute to its ongoing development by critically examining how its three integrated dimensions can be applied to real‐world contexts.

This study draws on community narratives following a bushfire event to explore how the three dimensions of the Occupational Justice PLUS model intersect in disaster recovery contexts. Specifically, the analysis examines how disruptions to occupational participation (doing) are intertwined with whose knowledge of recovery is heard and valued (knowing) and how affected individuals and communities are interpreted and represented within institutional and professional responses (being understood). In doing so, the study offers a practical example of how occupational, epistemic, and hermeneutic justice can be understood in post‐disaster contexts.

To examine the model in greater depth, we now outline its core components more explicitly. As described previously, Whiteford (2019) proposes three key foci for adopting a critical stance, calling for occupational therapists to bring a greater awareness to three types of justice: occupational justice, epistemic justice, and hermeneutic justice (see Figure 1). Rather than presenting these as isolated concepts, the model positions them as dynamically interconnected, such that injustice in one domain may reinforce or perpetuate inequities in the others. Clarifying these three foci provides a necessary foundation for understanding how the Occupational Justice PLUS model can be meaningfully applied in real‐world contexts.

**FIGURE 1:**
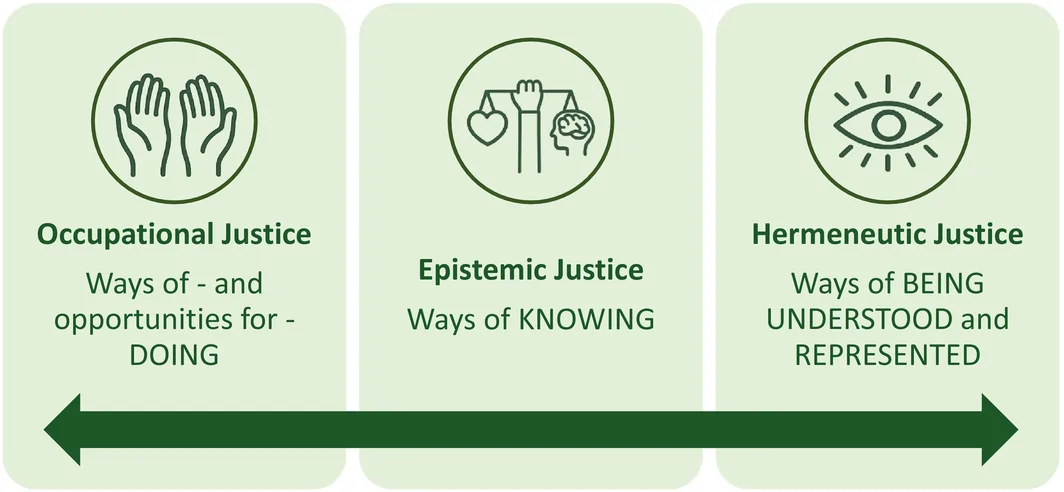
Occupational Justice PLUS.

### 1.1 Occupational justice: Ways of—and opportunities for—doing

In the first part of the Occupational Justice PLUS model, Whiteford (2019) describes the need to advance occupational justice by prioritising ways of—and opportunities for—doing. The importance of advancing occupational justice for individuals and populations has been well established in previous literature (Townsend, 1999; Townsend & Whiteford, 2005; Townsend & Wilcock, 2004; Whiteford, 2000; Wilcock, 2006) and can be defined as work towards the advancement of ‘economic, political, and social forces which create equitable opportunity and means to choose, organise, and perform occupations that people find useful or meaningful in their environment’ (Townsend, 1999, p. 154).

A commitment to occupational justice requires deliberative focus on mobilisation of resources with ‘the aim of creating occupationally “just” societies, societies based on people and their need, and indeed right, to do’ (Whiteford, 2000). Scholars and practitioners have described how tools such as the Participatory Occupational Justice Framework (Townsend & Whiteford, 2005) and the Capabilities, Opportunities, Resources and Environments—or CORE approach (Pereira, 2017) can promote occupational justice through focussed attention on broader structural arrangements compounding disadvantage, with implications for occupational therapy practice.

### 1.2 Epistemic justice: Ways of knowing

In the next part of the Occupational Justice PLUS model, Whiteford (2019) calls for epistemic justice—or a prioritisation of clients' knowledge. This builds on the concept of epistemic injustice as presented by Fricker (2007), who describes the harm that occurs when a person is insulted or wronged in their capacity as knower and is thereby restricted from their liberty to convey knowledge and make sense of their own social experiences.

A commitment to epistemic justice requires occupational therapists to acknowledge, solicit, value, and integrate our client's knowledge while recognising the need to undertake system‐level advocacy to ensure conditions are conducive to the valuing of such knowledge (Katzman & LeBlanc‐Omstead, 2019). For occupational therapy scholars and practitioners, awareness of our own prejudiced belief systems and epistemic reflexivity on the social conditions under which disciplinary knowledge has been produced is crucial (Farias et al., 2016; Kinsella & Whiteford, 2009; Leblanc & Kinsella, 2016).

### 1.3 Hermeneutic justice: Ways of being understood and represented

In the final part of the model, Whiteford (2019, p. 685) suggests that there is a need to enact hermeneutic justice, ensuring people ‘have rights of representation of their identity in a way that they are comfortable with, without fear of reprisal or vilification’. This approach aims to address hermeneutic injustice—the harm that is caused when significant areas of one's social experience are obscured from collective understanding as a result of the presence of wrongful interpretive obstacles (Fricker, 2007; Medina, 2013).

A commitment to hermeneutic justice is the ‘need to support people in their ability to be unconditionally self‐defining as they engage in a myriad of occupations on a daily basis in diverse contexts’ (Whiteford, 2019, p. 685). Occupational therapy practitioners and scholars will need to commit to working in a way that allows access and interchange between different knowledge systems, working in solidarity with community members to expand opportunities for all to enter into dialogue and contribute to collective knowledge within the profession.

### 1.4 Research aim

By drawing on narratives (written and film) from the 2019 bushfire affecting the community of Conjola on the New South Wales south coast (Australia), this study aims to explore how learnings from community members' responses to a bushfire event could broaden understanding of occupational, epistemic, and hermeneutic justice in the context of disasters and, more broadly, other injustices.

## 2 METHODS

### 2.1 Ethics

The data analysed for this study comprised a publicly available written narrative and a publicly available film, both produced by the community themselves, reflecting their voices, perspectives, and ways of making sense of the event. These materials were selected because they provide rich, multilayered accounts of lived experience, including emotional, social, and cultural dimensions, which are central to understanding the phenomena under study. Consistent with National Health and Medical Research Council (2025) guidelines on secondary use of data, no ethics application was required. While participants featured in the film and narrative did not provide explicit research consent, steps were taken to respect their privacy and dignity, including the removal of names and other identifying details. The study also considered the context in which the materials were produced and the potential risks associated with their use. These measures minimised potential harm and ensured that participants' anonymity and dignity were preserved throughout the study, while allowing the rich, multilayered accounts of community disaster recovery to inform understanding of occupational, epistemic, and hermeneutic justice.

### 2.2 Positionality statement

At the time of this study, the research team comprised four occupational therapy academics: one early‐career researcher and three experienced academics. All identify as cisgender, able‐bodied, White females and were living in metropolitan or regional Australian settings. The lead researcher's personal experience of disaster events, along with the team's professional backgrounds and expertise in occupational justice, shaped the focus of the research questions and influenced interpretation of the narratives, particularly the emphasis on occupation, health, and wellbeing. As a team, we recognise our privileged positions in communities where we are highly educated health professionals and hold status within dominant cultures. On balance, we also drew on our experiences working and living cross‐culturally and in rural regions across different Australian states to guide our approach to this project. Our positionalities were acknowledged, and reflexive practices were employed throughout the study to critically consider how the collective beliefs and experiences may have guided analytic choices and framing of findings. While our intentional efforts to limit epistemic and hegemonic blind spots have been a prominent feature of our discussion and reflection, we recognise the potential for limitations in our analysis.

### 2.3 Study design

In this exploratory qualitative study, a phenomenological hermeneutical method was used. This approach gave recognition to the understanding that ways of narrating an experience carry aesthetic, ethical, and political implications. They include, on the one hand, the different ways of saying about an utterance and, on the other hand, the perspectives of the narrator about the world and about themselves (Allegretti & Magalhães, 2022; Farias & Magalhães, 2022). Written and film narratives analysed for this study were interpreted as expressions of participants' life‐world phenomena, with analysis focussed on the experiential and existential significance of events, actions, and social processes. This method allowed researchers to develop an intersubjectively understandable interpretation of community experiences of occupational, epistemic, and hermeneutic justice (Lindseth & Norberg, 2022; Ricoeur, 1976).

### 2.4 Data collection and selection

The extant written narrative analysed for this research was from the publication *Doing Our Best: Individual and Community Responses to Challenging Times*, a project undertaken by Occupational Therapy Australia (2022) to capture the strategies employed by people during successive disaster events. The written narratives from Doing Our Best were reviewed in their entirety as part of a broader research project. However, this present analysis focussed on a single narrative sampled for its rich description of disaster experience and recovery. While the initial plan was to focus analysis on the selected narrative alone, we also sought to include the film ‘We are Conjola’ (produced by the narrative writer) as an extension of the primary dataset. With the depth of narrative limited by the length and scope of the original project, inclusion of the ‘We are Conjola’ film allowed for a more in‐depth exploration of community‐driven disaster recovery. While both sources informed all stages of analysis, the film served as the primary source of illustrative quotations due to its rich, first‐person community accounts, with the written narrative used to support theme development and deepen interpretive understanding. Therefore, both the written narrative from the *Doing Our Best* publication and the film served as sources for analysis.

### 2.5 Data analysis

The narrative and film were both analysed using a three‐step phenomenological hermeneutical approach as outlined by Lindseth and Norberg (2022) based on the work by Ricoeur (1976).

Data analysis was conducted by the lead researcher (E. H.), supervised by the co‐authors (C. M., G. W., and E. G.), all of whom had access to the written narrative and film. The analysis began with a naïve reading/viewing in which the whole narrative was read, and the film was watched to gain a general understanding of the phenomena studied (Lindseth & Norberg, 2022).

Once a naïve understanding had been formulated from both sources, a structural analysis was performed, in which meaningful units were identified, marked, and then condensed by rewriting their essential meaning in everyday words as concisely as possible (Lindseth & Norberg, 2022). These condensed units were sorted into subthemes and themes, which were then compared against the naïve understanding (Lindseth & Norberg, 2022). An example of this analysis is provided in Table 1. This process of comparison was repeated until the naïve understanding and the structural analysis validated each other and did not exclude any data, ensuring credibility and dependability of interpretations (Lindseth & Norberg, 2022).

**TABLE 1:** Examples of themes and subthemes.

Themes	Subthemes
Dissatisfaction with the government's emergency response	Despite significant previous research, the government was still not acting on climate change, so the probability of a major fire event was growing each year. Disbelief, sadness, and anger from the community over fire management orders that were made. The community felt abandoned during the fire and in the recovery period.
Dissatisfaction with the media depiction of the fire event	Personal stories sat behind the events of the fire but were not being told by mainstream media. The community was dissatisfied with the information portraying the causes of the fire. The community was aware of stories on the fire being quickly swept out of public consciousness by the 24/7 news cycle.

In the final stage of data analysis, a comprehensive understanding was formulated by the lead researcher through reading the whole text and the themes from the structural analysis. Then, under the supervision of the research team, they further critiqued the themes, reflecting on them in relation to the narratives, literature, and Occupational Justice PLUS model (Lindseth & Norberg, 2022). While the initial naïve reading and structural analysis were conducted inductively, the subsequent interpretive phase involved an abductive process in which themes were examined against the conceptual lenses of occupational, epistemic, and hermeneutic justice. All authors engaged in reflexive, iterative discussions throughout to challenge assumptions, enhance transparency, and confirm that interpretations were grounded in the narratives themselves. Where themes appeared to span more than one domain, this overlap was retained and explicitly discussed, consistent with the model's premise that these forms of injustice are interconnected rather than discrete. Detailed documentation of analytic steps, including examples of meaning units, subthemes, and themes, provided an audit trail to support confirmability of findings. An example of this analysis is provided in Table 2.

**TABLE 2:** Interpretation in relation to the Occupational Justice PLUS model.

Themes	Primary domain of justice	Secondary overlapping domain(s)
Aboriginal ways of knowing under‐utilised.	Epistemic justice	Hermeneutic justice
In recovery, it was the community who pitched in to help one another.	Occupational justice	‐
Through art, the community hopes to share learnings from this fire event with the rest of Australia.	Hermeneutic justice	Occupational justice, epistemic justice

Although the findings are presented under the domains of occupational, epistemic, and hermeneutic justice, these emerged inductively from the community's own narratives rather than being pre‐imposed. The analysis remained consistent with a phenomenological approach, focussing on the meaning and significance participants ascribed to their experiences, with the justice domains serving as a structuring lens to communicate insights. This approach preserves the richness and depth of lived experience while highlighting patterns of injustice in a way that is faithful to participants' voices.

## 3 FINDINGS

On New Year's Eve 2019, the community of Conjola on the New South Wales south coast (Australia) was impacted by a large‐scale bushfire. The fire resulted in the destruction of 114 homes in the area, destroyed approximately 70% of the surrounding bushland, and led to the loss of three lives. A local resident and filmmaker was interstate at the time and saw what was left of his home in the background of a TV news report. In the recovery period, the community, who had previously closed its doors to all media, entrusted this community member to create a film to tell their stories.

The naïve viewing of the narrative/film revealed the deep emotional impact caused by the event, with community members describing feelings of disbelief, sadness, anger, and fear. They also described a dissatisfaction with the way the fire recovery and media coverage were managed, expressing a desire both to uncover the causes of the fire and to tell their story of recovery in their own way. The film highlighted the strength of the community, who came together to support the immediate response to the fire and longer term recovery. The value of art was exemplified as both a form of healing and a means to communicate the impacts of the fire with the wider community.

Following naïve and structural analysis, the themes identified were interpreted and organised under three types of justice described by Whiteford (2019): occupational justice, epistemic justice, and hermeneutic justice. These headings are used for clarity, but they were derived from the lived experiences captured in the narrative and film, rather than imposed prior to analysis. Direct quotations presented in the findings are primarily drawn from the film, with the written narrative informing thematic development and interpretive depth.

### 3.1 Experiencing abandonment and taking action—Occupational justice

Community members impacted by the fire clearly experienced occupational injustice—because they were denied the physical, social, economic, or cultural resources or opportunities for engaging in necessary or meaningful occupation (Carra et al., 2019). In the immediate emergency response phase of the fire, community members described how a lack of resources left them feeling abandoned:

Getting to the end of our road and having fire trucks drive past me and I'm out of the car screaming that my house was on fire and my husband's inside and nobody stopped. I'll never get over that.

In the days following the fire, residents were restricted from getting back into the community and thus felt a sense of helplessness:

There was a roadblock put up at the highway at the turnoff into Lake Conjola. And once you went through that roadblock, you weren't allowed back in. So effectively, the community of Conjola Park, the Lake Conjola and others were isolated for eight days.

In the period of recovery, the lack of civil response compounded the community's perceived sense of abandonment:

That lack of a civil response left me a little stunned. I was quite angry about it very early. That we had no one come. That the police didn't come, the Council didn't come. The SES didn't come. No one came. But we were abandoned. Completely abandoned. Not one RFS or National Parks and Wildlife service or fire and rescue person came to say, are you alright? Is there anything that we could do now, even if we didn't do it then?

With the community experiencing a lack of support from government emergency response teams, it was residents themselves who felt a shared sense of responsibility to help one another. Community‐driven action was crucial to the restoration of occupational justice. When discussing how fellow community members were ready to jump in to help one another during the fire event, one resident said: ‘The wonderful thing about it was that the local saved locals.’ As they moved through the recovery phase, assets within the community began to emerge:

…. The thing that I'll never forget is the talent that came forward, the skills that people had and the effort that people were prepared to put in ….

It was clear that the community was proud of the way they came together to support one another and restore occupational justice:

Out of the recovery have come some of the most beautiful things and things that I probably will remember way more than the day itself.

### 3.2 Claiming voice and knowledge—Epistemic justice

Community dissatisfaction with the government moved beyond anger at the emergency response to include the pervasive lack of action on bushfire preparedness, despite growing community awareness of the role of climate change in increasing disaster risk. A clear description of epistemic injustice was evident.

I've been studying climate change since the 1990's. And knowing that governments have never done anything about it significantly, at all, ever and it's just getting worse. I knew the probability of me being hit by a bushfire just was going to get greater and greater every year.

Community members also raised issues with the way the government had ignored experts in climate change despite the issue of increasing fire risks being raised:

Emergency Leaders for Climate Action Group petitioned the government, in an open letter, we asked to meet with the prime minister and we also asked for the urgent release of funds for additional aerial firefighting aircraft. And we wanted more research undertaken into the nature of the extreme bushfires that we're having …. The outcome was certainly not a discussion with the prime minister. Initially we were pushed to one side and in fact, there were many attempts to discredit the group …. Climate change is a dirty word in Australia. And again, that's been something that, you know, the major parties have absolutely polarised and politicised with their friends in the Murdoch media.

The community was similarly upset by the government's disregard of Aboriginal ways of knowing and under‐utilisation of traditional land management practices that may have reduced the risk of large‐scale bushfire events:

Cultural burning. Why is it important to Indigenous culture? Well I believe it should be important to Australian culture …. We burn the land. But we burn the land only when the conditions are right and only in the places where it needs to be burned. You don't just indiscriminately set fire to something and burn it.

With community members frustrated with the government's inaction on bushfire preparedness and their politicisation of climate change as an issue, the community was also concerned about the media's role in addressing the issue:

Conjola and other communities that were torn apart by last summer's bushfires will be left alone to rebuild. All mostly forgotten. Swept out of public consciousness by the 24/7 news cycle.

The community of Conjola felt wronged in their capacity as knowers, as they were restricted from opportunities to convey knowledge and make sense of their own social experiences. In response to this epistemic injustice, they felt a strong sense of responsibility to share their story their way through the creation of the ‘We Are Conjola’ film:

…. This isn't my story. This is the story of a deeply scarred yet inspiring community looking for answers. And how they created their pathway to recovery.

The film itself became a vehicle to restore epistemic justice, a way for the community to convey learnings from the Conjola bushfire to unpack and potentially prevent mismanagement of emergency response and recovery occurring again:

There is one question that we need to act on. How can we stop this from happening again? There are answers, but they lead to another question. Do we as a global community have the courage to do what needs to be done …?

### 3.3 Expressing identity and community through art—Hermeneutic justice

Before the disaster, Lake Conjola residents identified as a community of artists. As the community sought to address the harm that was caused when significant areas of their social experience were obscured from collective understanding (hermeneutic injustice), art became the vessel the community used to express themselves, their stories, and their frustrations with government inaction on climate change and disaster preparedness. One resident's poem provides a poignant summary of community sentiment:

So the poems are just a part of the phoenix arising from our now empty blocks, but our hearts are full and we're having a conversation longer, more in depth than ever before.

We'll write on, paint and make documentaries, never to forget that torment, hoping a lesson has been learned, funding in place to face the next onslaught.

The government departments won't get it right. Of course, it's in their DNA to represented vested interests, not us, sorry to say.

But our carefully considered work might just give them a push in the right direction, reminding them of who they should represent.

So, let's get on with art in its many forms, so that, as famously said, let a thousand flowers blossom.

Seeking representation of their identity in a way that they were comfortable with (hermeneutic justice), the community very purposefully used art in its many forms to share learnings from the bushfire event:

Since the fires. My writing has changed. It's intensified. And I think I go to a deeper place when I'm writing. I'm processing things that I experienced immediately, very viscerally. I feel that there's a greater need now to get my work out, to get it published and to get it into the wider sphere so that people in the rest of Australia can understand what it's like to experience a bushfire. Because this is going to happen again, and no one is safe anymore.

For others in the Conjola community, music, paintings, and sculptures became the medium that allowed for the interchange between different knowledge systems—a way for the community to share their lived experience with the wider Australian community. The film, funded by the community itself, also stood as an important art piece used to restore hermeneutic justice. The community strongly expressed their desire for the film to act as a historical document from which government agencies could learn from their failures, to lessen the risk and impact of such disasters in the future.

## 4 DISCUSSION

This study aimed to explore how learnings from community members' responses to a bushfire event could broaden our understanding of occupational, epistemic, and hermeneutic justice in the context of disasters and, more broadly, other injustices. The analysis highlighted occupational, epistemic, and hermeneutic injustices experienced by community members impacted by the Conjola bushfire. The findings of this study invite consideration of how occupational therapists might use these three key foci to adopt a critical stance, specifically in the field of disaster management but also in practice more widely.

In relation to the theme of occupational justice, community members described occupational injustice—being denied the physical, social, economic, or cultural resources or opportunities for engaging in necessary or meaningful occupation (Carra et al., 2019). For many, government decisions related to emergency response limited the availability of physical resources to perform critical self‐care occupations, resulting in feelings of helplessness, abandonment, and trauma. Consistent with previous research exploring restoration of occupational justice post disaster, it was community‐driven action that supported recovery (Carra, 2014; Carra et al., 2019; Huppatz et al., 2025; Rushford & Thomas, 2016). The community drew on its assets and strengths and gained a sense of pride for what was accomplished collectively. For occupational therapists with an interest in supporting restoration of occupational justice in disaster recovery, models and theories that support strengths‐based community‐driven action (such as the Participatory Occupational Justice Framework) may be particularly valuable.

The findings illuminated a clear description of epistemic injustice, both in the way the government had disregarded key voices raising concerns over the increasing risks of climate‐related disasters and in the way they had under‐utilised Aboriginal ways of knowing relating to cultural burning.

In the wider literature, the importance of community partnership and engagement in disaster preparedness and emergency response was highlighted by Nibanupudi and Choudhury (2015), explaining that in the Indian subcontinent, where disaster response agencies consulted and engaged communities on appropriate action and relief, damage from disasters was reduced and responses were more comprehensive. In Canada, research showed that responding to wildfires can be a shared responsibility between communities and institutions but that this requires trust and actively integrating local knowledge into response planning (van Kerkhof & de Almeida Serranito, 2025). In Japan, much of the focus of disaster risk reduction has been on the built environment, but a study by Kawane et al. (2024) showed that social innovation (e.g., participatory community engagement, cross‐sector stakeholder collaboration, and inclusive approaches for vulnerable populations) can help to mitigate risks and enhance community resilience.

For the community in Conjola, their anger compounded their trauma as they felt wronged in their capacity as knowers. Frustration over media representation of the event caused further harm as the community was restricted from their liberty to convey knowledge and make sense of their own social experiences. In the face of these challenges, the community rallied to tell their story their way, to share their learnings from the bushfire, and to prevent potential mismanagement of future disasters. This intent appeared to hold therapeutic benefit, as the community gained a sense of redress of justice. For occupational therapists, utilisation of narrative approaches that privilege community voice (such as the CORE approach) may support empowerment by recognising community experience and expertise and promoting agency. Recognising that stories are an important part of effective advocacy for systemic change, occupational therapists may also play a role in amplifying community narratives across all levels of government.

The community's experiences reflected instances of hermeneutic injustice, in which significant aspects of their social realities were obscured from broader collective understanding. In response, they engaged collectively in artistic practices, serving both as a shared pathway to healing and as a means of representing their identities on their own terms, thereby fostering hermeneutic justice. These practices spanned a wide range of creative forms, including writing, poetry, visual art, sculpture, filmmaking, and music. The role of arts in community development is not new, but our critique amplifies the way narrative and film can underscore the importance of adopting creative approaches that facilitate interchange between diverse knowledge systems. This requires partnering with community members to support their active participation in dialogue to ensure that their collective knowledge and lived experiences meaningfully inform professional practice. This is consistent with trends in occupational therapy and inter‐professional practice utilising codesign (Auger et al., 2025; Kehoe et al., 2025; Layton et al., 2024) and participatory approaches in community settings (Anzai et al., 2026; Cowen et al., 2024; Hyett et al., 2019; Irvine‐Brown et al., 2021).

Importantly, the findings demonstrate that occupational, epistemic, and hermeneutic injustices are not discrete but mutually reinforcing (Whiteford, 2019). Restricted access to material and social resources (occupational injustice) constrained opportunities for communities to assert and share their knowledge (epistemic injustice), whereas misrepresentation or narrative invisibility (hermeneutic injustice) further limited their capacity to influence systems affecting occupational participation. These intersecting dynamics suggest that addressing one dimension of injustice in isolation is insufficient. Whiteford's (2019) Occupational Justice PLUS model therefore offers a holistic lens through which occupational therapists can identify and respond to systemic, knowledge‐based, and representational inequities simultaneously.

Beyond its practical application, we argue that the model also serves to guide critical consciousness regarding gaps within dominant knowledge systems. The findings illustrate how disaster discourse frequently privileges institutional, governmental, and media narratives while marginalising community knowledge, Aboriginal knowledge systems, and lived experience of community members. By foregrounding epistemic and hermeneutic justice alongside occupational justice, the Occupational Justice PLUS model directs attention to whose knowledge is legitimised, whose experiences are rendered visible, and whose realities are excluded from policy and recovery planning. In doing so, it invites occupational therapists not only to intervene at the level of service provision but also to interrogate the epistemological foundations of disaster management and of the profession itself. The model thus functions as a critical tool for identifying systemic silences and advocating more inclusive approaches to knowledge production.

Collectively, these findings highlight not only the presence of occupational, epistemic, and hermeneutic injustices but also the ways in which communities actively mobilise to redress these injustices. The Occupational Justice PLUS model offers a conceptual lens through which occupational therapists can critically examine such dynamics.

### 4.1 Implications for practice

The findings illustrate how the Occupational Justice PLUS model moves beyond theoretical abstraction to guide meaningful action, advocacy, and capacity‐building within the occupational therapy profession. We propose that in future practice, occupational therapists may use the model to guide intervention design in disaster‐affected communities, focussing on enabling meaningful occupations, recognising local knowledge, and supporting authentic representation of community experiences. Creative and participatory methods can serve simultaneously as therapeutic and advocacy tools, helping communities communicate experiences, assert knowledge, and shape systemic responses.

In research, the model offers a framework for investigating the interplay between different forms of justice across populations, contexts, and events. By privileging participant narratives and community expertise, future studies can examine how interventions foster or hinder justice and generate evidence to guide policy and systemic change.

### 4.2 Study strengths and limitations

Given the heightened vulnerabilities of communities affected by disaster, the use of publicly available media represents a methodological strength of this study. This approach allowed access to rich, first‐hand narratives of recovery and resilience while minimising potential risks of traumatisation or undue burden on participants.

However, several limitations warrant consideration. First, the written narrative and film were produced for purposes other than research. As secondary data sources, they did not allow opportunities for clarification, probing, or expansion of emerging ideas. The perspectives presented were therefore shaped by the intentions of the original creators and the constraints of media production, which may have limited the breadth and depth of issues explored. The use of two distinct data forms also introduced interpretive complexity, as each medium affords different forms of expression and emphasis.

Second, although the initial structural analysis was conducted inductively, interpretation was subsequently undertaken in relation to the Occupational Justice PLUS model. While this theoretically informed approach strengthens conceptual coherence, it may also have directed analytic attention towards instances of occupational, epistemic, and hermeneutic injustice, potentially constraining alternative interpretations. The risk was mitigated through iterative dialogue among all authors and by retaining themes that demonstrated overlap across domains, consistent with the model's integrative premise.

Finally, the study reflects interpretation of a single community's experience within a specific disaster context. As such, findings are not intended to be generalisable but rather to offer conceptual insights that may inform critical reflection and application in other contexts.

## 5 CONCLUSION

This study illuminates community members' experiences of occupational, epistemic, and hermeneutic injustice in the context of a bushfire event. It also demonstrates the significance of community‐driven, narrative‐based initiatives in addressing these injustices. By taking control of their own narratives, the Conjola community was able to foster healing while actively contributing to the prevention of similar shortcomings in government responses in future disaster responses. These findings offer valuable insights for the occupational therapy profession.

## AUTHOR CONTRIBUTIONS

**Eliza Huppatz**: Conceptualisation; formal analysis; methodology; writing—original draft; writing—review and editing. **Carol McKinstry**: Conceptualisation; formal analysis; methodology; writing—original draft; writing—review and editing. **Gail Whiteford**: Conceptualisation; formal analysis; methodology; writing—original draft; writing—review and editing. **Emma George**: Conceptualisation; formal analysis; methodology; writing—original draft; writing—review and editing.

## CONFLICT OF INTEREST STATEMENT

The authors declare no conflicts of interest.

## Data Availability

The data that support the findings of this study are available from Occupational Therapy Australia. Restrictions apply to the availability of these data, which were used under licence for this study. Data are available from the author(s) with the permission of Occupational Therapy Australia.
